# Risks of Development of COVID-19 among Patients with Inflammatory Bowel Disease: A Comparative Assessment of Risk Factors for Incident Infection

**DOI:** 10.1093/crocol/otac011

**Published:** 2022-03-29

**Authors:** Millie D Long, Xian Zhang, James D Lewis, Gil Y Melmed, Corey A Siegel, Emily Cerciello, Angela Dobes, Alandra Weaver, Laura Weisbein, Michael D Kappelman

**Affiliations:** University of North Carolina, Department of Medicine, Division of Gastroenterology and Hepatology; Center for Gastrointestinal Biology and Disease, University of North Carolina, Chapel Hill, NC; Center for Gastrointestinal Biology and Disease, University of North Carolina, Chapel Hill, NC; University of Pennsylvania, Department of Medicine, Division of Gastroenterology and Hepatology, Philadelphia, PA; Cedar Sinai Medical Center, Los Angeles, CA; Dartmouth-Hitchcock Medical Center, Lebanon, NH; Crohn’s & Colitis Foundation, New York, NY; Crohn’s & Colitis Foundation, New York, NY; Crohn’s & Colitis Foundation, New York, NY; Center for Gastrointestinal Biology and Disease, University of North Carolina, Chapel Hill, NC; Center for Gastrointestinal Biology and Disease, University of North Carolina, Chapel Hill, NC; University of North Carolina, Department of Pediatrics, Division of Gastroenterology and Hepatology

**Keywords:** IBD, Crohn’s disease, ulcerative colitis, COVID-19, SARS-CoV-2

## Abstract

**Background:**

Patients with inflammatory bowel disease (IBD) may be at risk for development of COVID-19 infection due to innate immune dysfunction and/or immunosuppressive medication use.

**Methods:**

In a prospective cohort of adult IBD patients, we captured data on clinical risk factors and IBD medication utilization. The outcome of interest was development of patient-reported laboratory confirmed COVID-19. We calculated incidence rate and performed bivariate analyses to describe the effects of risk factors (age, immunosuppression use, obesity, race) on development of COVID-19. We utilized logistic regression models to determine the independent risks associated with each factor.

**Results:**

A total of 3953 patients with IBD were followed for a mean duration of 212 days (SD 157). A total of 103 individuals developed COVID-19 during follow up (2.6%, rate of 45 per 1,000 person-years). Severity of infection was generally mild. Clinical characteristics were similar among those who developed COVID-19 as compared to not. African American race was associated with incident COVID-19 infection (OR 3.37, 95% CI 1.18-9.59).Immunosuppression use was not associated with development of COVID-19 (OR 1.19, 95% CI 0.72-1.75), nor was age (OR 1.00, 95% CI 0.99-1.02), nor obesity (OR 1.01, 95% CI 0.61-1.66).

**Conclusions:**

Immunosuppression use did not increase the risk of development of COVID-19. Therapeutic management of IBD should not be altered to prevent a risk of developing COVID-19.

Introduction:

As the COVID-19 pandemic has progressed, researchers now have a better understanding of the risk factors for development of severe COVID-19 complications among those infected with SARS-CoV-2. One such risk factor is use of immunosuppressant medications, including corticosteroids. Other risk factors include older age and comorbidities.[1,2] A population that may be at increased risk for COVID-19 complications is those with inflammatory bowel disease (IBD), including both Crohn's disease (CD) and ulcerative colitis (UC). These chronic inflammatory conditions of the gastrointestinal tract affect millions of people worldwide. [3-5] Individuals with IBD often require ongoing immunosuppressive medication use. Therefore, it is possible that individuals with IBD may be at increased risk for both 1) development of COVID-19 and 2) progression to severe COVID-19.

The international SECURE-IBD registry[6] has helped to define the medication-specific risks of severe complications from SARS CoV-2 infection. Patients treated with corticosteroids or anti-tumor necrosis factor alpha (anti-TNF) agents combined with immunomodulators and those on thiopurines have increased risks of severe infection.[6,7] However, as all individuals in the SECURE-IBD registry have a diagnosis of COVID-19, the incidence of and risk factors for COVID-19 infection cannot be determined. Less is known about the rate of development of COVID-19 infection among individuals with IBD, particularly among those that do not require hospitalization. This is due to difficulties capturing diagnosis of non-hospitalized COVID-19 infection in an ambulatory population where there is a known population denominator. Diagnosis of COVID-19 infection is also elusive in administrative claims data, due to widespread availability of home and free testing in the United States. Therefore, patients with IBD, and their physicians, face a **critical knowledge gap** regarding risk factors for developing COVID-19.

Prior qualitative work has demonstrated the overwhelming concern felt by IBD patients in regards to risks of developing COVID-19.[8] Patient inquiries to the Crohn's & Colitis Foundation, the major advocacy organization for IBD patients, has skyrocketed over the pandemic, signaling a need for patient-centered comparative safety research to understand the risk factors associated with development of COVID-19 in the IBD population. To address this need, we conducted a prospective direct-to-patient cohort of individuals with IBD to determine the incidence of COVID-19 infection and medication specific attributable risks.

Methods:

We performed a prospective cohort study of adult (age ≥18) patients with inflammatory bowel disease to understand rates and outcomes of COVID-19 infections from April 23, 2020 until August 30, 2021. Patients in 3 separate studies sponsored by the Crohn's & Colitis Foundation, IBD Partners, IBD QORUS (Improving the Quality of Care for Adults with Inflammatory Bowel Disease), and SPARC IBD (Study of a Prospective Adult Research Cohort with IBD), were included in this study. Details surrounding development of each of these cohorts are published elsewhere.[9-11] Participants received online surveys with questions on comorbidities, medication utilization and development of COVID-19 at baseline, 2, 4, 6, 8 weeks later, and then every 6 months. For those in the SPARC IBD cohort, IBD diagnosis and medication utilization was verified by medical records and pharmacy data. Participants from the IBD Partners cohort were also asked questions on adherence to public health recommendations for avoidance of COVID-19, such as social distancing. Initial recruitment occurred between April 23, 2020 and August 25, 2020, with data collected through August 30, 2021.

Exposures of interest included comorbidities, age, race, obesity, CD vs. UC diagnosis, and medication utilization. Age was defined in a total of 3 categories (18-34 years, 35-65 years and >65 years) or continuously.   Obesity was defined as a body mass index ≥ 30.  Race was defined as African American vs. other (predominantly Caucasian) and medication utilization was defined a priori as any immunosuppressive medication use vs. none (including corticosteroids, biologics, JAK inhibitors, thiopurine analogues and methotrexate).

The primary outcome was COVID-19 infection, defined as a self-reported COVID-19 infection with confirmation by PCR and/or antigen testing testing . For each active infection, the date, type of test (antigen, PCR), route of testing (nasal swab, saliva) and test results were collected. Only those individuals who reported a positive PCR or antigen test were considered to have had COVID-19 infection for this analysis. We did not include those with only serum antibody testing positive for the SARS-CoV2 nucleocapsid. We also captured information on those who could not obtain testing early in the pandemic, but were told by a healthcare provider that they probably had COVID-19 based on symptom complex.  For those who developed COVID-19 infection, self-reported severity of illness was obtained using a likert scale: very mild, mild, moderate, severe or very severe.

Participant follow-up was censored at the time of COVID-19 diagnosis or last known follow up as COVID-19 free.  Those who entered the cohort with a prior or current diagnosis of COVID-19 were excluded from the analysis.

Data analysis

Clinical characteristics and outcomes were defined within each individual cohort (IBD Partners, SPARC and QORUS) to ensure compatibility, prior to combining data into the overall cohort. Bivariate analyses were performed for each exposure by development of COVID-19 infection. *A priori* selected factors included in logistic regression models to determine independent risk factors for COVID-19 infection included age, race, obesity and immunosuppression use. All analyses were performed in SAS, Cary, NC. The study protocol was approved by the institutional review board of the University of North Carolina, University of Pennsylvania and Dartmouth-Hitchcock Medical Center

Results:

A total of 3953 patients with IBD were included in the overall cohort, 2541 with CD and 1354 with UC or indeterminate colitis. A majority of the cohort were on biologic and/or immunomodulator therapy at inclusion. The mean follow up time for the cohort was 212 days (SD 157) (Table 1).

A total of total, 103 individuals in this population developed COVID-19 (2.6%). When we included those individuals who reported likely COVID-19 infection confirmed by a provider, but no positive test due to limited testing capabilities early in the pandemic (n=25), the total number of cases remained comparable [n=128 (3.2%)]. When calculated in person-years, the rate of lab confirmed COVID-19 infection was 45 per 1,000 person-years for the overall IBD cohort. Characteristics of those who developed COVID-19 as compared to those who did not were similar, with somewhat higher rates of CD diagnosis (73% vs. 65%, p=0.10), pre-existing chronic pulmonary conditions (21% vs. 16%, p=0.17), and African American race (4% vs. 1%, p=0.02) among COVID-19 positive patients. Obesity was not associated with COVID-19 infection in the IBD population, nor was disease activity, nor immunosuppressive medication use (Table 2). Of those who developed COVID-19, 19.1% self-identified their infection as very mild, 32.0% mild, 42.7% moderate, 5.8% severe, 0% very severe (Figure 1).

In multivariable models, use of immunosuppression use was not associated with development of COVID-19 (OR 1.12, 95% CI 0.72-1.75), nor was age (OR 1.00, 95% CI 0.99-1.02), nor obesity (OR 1.00, 95% CI 0.61-1.66). African American race vs. other was associated with development of COVID-19 in this IBD population (3.37, 95% CI 1.18-9.59) (Table 3)

In a sub-analysis of the IBD Partners cohort (n=2168), the majority reported a high rate of concern surrounding COVID-19 infection (54% extremely or moderately concerned). The population had high adherence to public health measures to protect against COVID-19, with 98% adhering to social distancing, 97% hand washing, 64% self-isolation, 56% working from home and 87% limiting social activities. Level of concern was significantly associated with development of COVID-19 infection, with fewer COVID-19 infections among those who were moderately or extremely concerned (p=0.04) (Figure 2).

Discussion:

In this large, prospective cohort of patient with IBD in the US, we determined the incidence of and risk factors for COVID-19 infection. This cohort also allowed for determination of risk factors for development of COVID-19. Importantly, immunosuppressive medications were not associated with an increased risk of COVID-19 infection (OR 1.12, 95% CI 0.72-1.75). Other studies of incident COVID-19 infection in IBD have been limited by sample size,[12] or administrative definitions of COVID-19 infection.[13] As patients in the US can receive testing in a number of sites, including home testing, pharmacy-based, state health department-based or hospital/clinic based, these tests are not always billed to insurance or recognizable within a health system's data. Therefore, it is possible that administrative studies of COVID-19 infection are associated with misclassification and measurement bias, in particular these studies may under count cases of COVID-19. For example, in a large study of

5303 IBD patients utilizing Partners Healthcare data, only 39 (0.7%) developed COVID-19.[13] This incidence is markedly less than that of our cohort (2.6%), suggesting under ascertainment. Nevertheless, this study also showed that immunosuppressive medications do not increase the risk of development of COVID-19. In a Veterans Affairs (VA) cohort, over a median follow up of 10.7 months, 649 patients (2.1%, 24.3/1000 person year) were diagnosed with COVID-19 infection. Data from single healthcare systems (such as the VA) do not include testing that happens elsewhere, likely underestimating the actual number of cases. In this VA cohort, patients on vedolizumab (OR HR 1.70, 95% CI 1.16-2.48) as compared to mesalamine had increased risk of developing COVID-19 infection.[14] The authors hypothesized that by binding to α4β7, vedolizumab could also affect the upper respiratory tract and thus be a risk factor for COVID-19.[14] There was no association between vedolizumab and development of COVID-19 in our study.

In rheumatoid arthritis (RA), another immune mediated disease (IMID), a higher rate of COVID-19 infection was observed as compared to age-matched non-RA patients in the VA system (incidence rate (IR) for RA of 27.3 (25.5, 29.2) per 1000 person-years).[15] The incidence rate in our IBD population was comparable to this estimate (45 per 1000 p-y), though he slightly higher rate we found in IBD may be related to how we captured positive COVID-19 diagnoses from the patient, including those from at-home or alternate testing.

In a separate meta-analysis of several IMIDs, there was a higher prevalence of COVID-19 as compared to general population, but of IMIDs, IBD had the lowest rate. This meta-analysis was limited due to heterogeneity.[16] The rationale for higher rates of COVID-19 infection in IMID populations is postulated to be related to corticosteroid use. As IBD is predominantly diagnosed at young ages, in individuals without significant other comorbidities, these factors may have influenced the rate of COVID-19 infection we found in IBD.

In our study, we found that African American race was independently associated with risk of incident COVID-19 infection. African American race has also been associated with both increased mortality and disease severity from COVID-19 infection in adults and children in a number of other studies.[17-20] Socioeconomic factors may play a role in the rates of COVID-19 infection in these populations, with increased exposures in individuals who work outside the home. However, rates of worsening outcomes such as hospitalization for COVID-19 in African Americans have been high regardless of poverty level.[20]

There are a number of strengths to this study of incident COVID-19 in IBD patients. The cohort is large, with a geographically diverse participation from across the US, including urban and rural areas. While the outcome was captured by self-report, we were able to obtain detailed information on type of testing in order to include only those with positive PCR or antigen testing. This is the preferred method of ascertaining COVID-19 status, as test results are not necessarily included in medical records or in administrative claims data due to the widespread availability of free testing. Another strength is that the participants from QORUS and SPARC IBD have confirmed IBD from medical records. There are also a number of limitations to this prospective cohort. For IBD Partners participants, diagnoses of IBD are by self- report, allowing for the possibility of misclassification. However, a prior validation study of a subset of IBD Partners showed that >97% of participants have confirmed IBD in medical records. Additionally, this was a convenience sample of patients who are highly engaged in research and had access to the internet so external generalizability may be limited. It is also possible that this group may have fewer occupational risk factors for COVID-19 related to higher socioeconomic status. We do not have access to specific zip code data, to determine whether urban/rural status affected the outcome. Additionally, we did not have access to vaccination status within this cohort. We started data collection early in the pandemic, prior to the availability of vaccines. COVID-19 vaccinations have demonstrated excellent efficacy at prevention of severe complications from COVID-19, although may not protect fully against development of COVID-19, which is the outcome of interest in this study. Finally, although large, our cohort did not have adequate power to investigate the independent risks of each class of medication used in IBD treatment.

In conclusion, rates of COVID-19 infection are low in patients with IBD and comparable to other IMID populations. This low rate is likely related to adherence to public health guidance among members of our cohort. These data are important to guide treatment paradigms in IBD patients, where continued maintenance therapies are needed to prevent relapse. These data provide reassurance that therapeutic regimens for IBD, including immunosuppressive agents, do not increase the risk of COVID-19 infection in IBD.

Data Availability:

Data for this manuscript include clinical data from the IBD Partners, SPARC and QORUS cohorts. These data are available through the Crohn's and Colitis Foundation as part of PLEXUS. Requests for data access should be made to PLEXUS through the following link: https://www.crohnscolitisfoundation.org/research/grants-fellowships/ibd-plexus

**Table 1: Characteristics of the Cohort of Patients with Inflammatory Bowel Disease**

| **Characteristics** | **N=3953** | **% or mean (SD)** |
|---|---|---|
| Age (years) | 3953 | 47.3 (15.3) |
| IBD type | | |
| Crohn's disease | 2541 | 65% |
| Ulcerative colitis/indeterminate colitis | 1354 | 34% |
| Gender (% female) | 2698 | 69% |
| Comorbidities | | |
| Chronic lung disease (asthma, COPD) (% yes) | 650 | 16% |
| Diabetes (% yes) | 176 | 4% |
| Kidney disease (% yes) | 118 | 3% |
| History of organ transplantation (% yes) | 16 | 0% |
| Active cancer (% yes) | 59 | 1% |
| Education (% >high school) | 2935 | 95% |
| Race (%) | | |
| Caucasian/other | 3903 | 99% |
| African American | 50 | 1% |
| Current smoking (% yes) | 83 | 4% |
| Obesity (BMI ≥30) (% yes) | 733 | 19% |
| Medications at enrollment (% yes)* | | |
| Anti-TNF | 1481 | 38% |
| Vedolizumab | 461 | 12% |
| Ustekinumab | 435 | 11% |
| Immunomodulator (IMM)* | 843 | 22% |
| Combination therapy (anti-TNF + IMM) | 389 | 10% |
| Corticosteroids (systemic) | 384 | 10% |
| Tofacitinib | 56 | 2% |
| 5-ASA[&] | 943 | 24% |
| Any immunosuppression (% yes) | 2782 | 70% |
| Duration of follow-up (days) | 3953 | 211.6 (156.9) |

*Anti-TNF (anti tumor necrosis factor alpha), IMM and combination groups not mutually exclusive

**methotrexate, azathioprine or 6-mercaptopurine

**Table 2: Characteristics of the Cohort by Development of COVID-19 Infection**

| Characteristics | COVID-19 + n=103 | | COVID-19 – n=3850 | | p value |
|---|---|---|---|---|---|
| | n | % or mean (SD) | n | % or mean (SD) | |
| Age (years) | 103 | 47.7 (13.2) | 3850 | 47.3 (15.3) | 0.78 |
| IBD type | | | | | |
| Crohn's disease | 75 | 73% | 2466 | 65% | 0.10 |
| UC/IC | 28 | 27% | 1326 | 34% | |
| Gender (% female) | 77 | 75% | 2621 | 69% | 0.17 |
| Comorbidities | | | | | |
| Chronic lung disease (asthma, COPD) (% yes) | 22 | 21% | 628 | 16% | 0.17 |
| Diabetes (% yes) | 3 | 3% | 173 | 4% | 0.44 |
| Kidney disease (% yes) | 3 | 3% | 115 | 3% | 0.97 |
| History of organ transplantation (% yes) | 0 | 0% | 16 | 0% | 0.51 |
| Active cancer (% yes) | 1 | 1% | 56 | 1% | 0.68 |
| Education (% >high school) | 83 | 92% | 2852 | 95% | 0.26 |
| Race (%) | | | | | |
| Caucasian/other | 99 | 96% | 3804 | 99% | 0.02 |
| African American | 4 | 4% | 46 | 1% | |
| Current smoking (% yes) | 1 | 1% | 82 | 4% | 0.26 |
| Obesity (BMI ≥30) (% yes) | 20 | 19% | 713 | 19% | 0.82 |
| Medications (% yes)* | | | | | |
| Anti-TNF | 43 | 42% | 1438 | 38% | 0.42 |
| Vedolizumab | 9 | 9% | 452 | 12% | 0.30 |
| Ustekinumab | 14 | 14% | 421 | 11% | 0.47 |
| Immunomodulator (IMM)** | 20 | 19% | 823 | 22% | 0.59 |
| Anti-TNF + IMM | 9 | 9% | 380 | 10% | 0.70 |
| Corticosteroids | 8 | 8% | 376 | 10% | 0.48 |
| Tofacitinib | 0 | 0% | 56 | 2% | 0.21 |
| 5-ASA[&] | 27 | 26% | 916 | 24% | 0.62 |
| Any immunosuppression (% yes) | 75 | 73% | 2707 | 70% | 0.58 |
| Duration of follow-up (days) | 103 | 291.2 (125.1) | 3850 | 209.5 (157.1) | <0.001 |

*Anti-TNF (anti tumor necrosis factor alpha), IMM and combination groups not mutually exclusive

**methotrexate, azathioprine or 6-mercaptopurine

**Table 3: Multivariate analyses of factors independently associated with development of COVID-19 Infection**

| Factor | Odds ratio* | 95% Confidence Interval |
|---|---|---|
| Any immunosuppression (yes/no) | 1.12 | 0.72-1.75 |
| Age (continuous) | 1.00 | 0.99-1.02 |
| Obesity (BMI≥ 30 vs <30) | 1.00 | 0.61-1.66 |
| African American race (vs. other) | 3.37 | 1.18-9.59 |

*Logistic regression model including age, obesity, immunosuppression, race

**Figure Legends**

**Figure 1: Self-reported Severity of COVID-19 Infection Among Patients with Inflammatory Bowel Disease**

*Note: no patient reported a very severe course of COVID-19

**Figure 2: Level of Concern among Inflammatory Bowel Disease Patients by COVID-19 Infection Status**

Figure 1

Figure 2

## Lay Summary

A total of 2.6% of IBD patients in this study developed COVID-19 infection. The rate of lab confirmed COVID-19 infection was 45 per 1,000 people over the course of a year. Infections were generally mild. Immunosuppressive medications did not increase the risk of developing COVID-19.

